# Large head metal-on-metal cementless total hip arthroplasty versus 28mm metal-on-polyethylene cementless total hip arthroplasty: design of a randomized controlled trial

**DOI:** 10.1186/1471-2474-9-136

**Published:** 2008-10-08

**Authors:** Wierd P Zijlstra, Nanne Bos, Jos JAM van Raaij

**Affiliations:** 1 Department of Orthopaedic Surgery, Martini Hospital, PO Box 30.033, 9700 RM Groningen, the Netherlands

## Abstract

**Background:**

Osteoarthritis of the hip is successfully treated by total hip arthroplasty with metal-on-polyethylene articulation. Polyethylene wear debris can however lead to osteolysis, aseptic loosening and failure of the implant. Large head metal-on-metal total hip arthroplasty may overcome polyethylene wear induced prosthetic failure, but can increase systemic cobalt and chromium ion concentrations. The objective of this study is to compare two cementless total hip arthroplasties: a conventional 28 mm metal-on-polyethylene articulation and a large head metal-on-metal articulation. We hypothesize that the latter arthroplasties show less bone density loss and higher serum metal ion concentrations. We expect equal functional scores, greater range of motion, fewer dislocations, fewer periprosthetic radiolucencies and increased prosthetic survival with the metal-on-metal articulation.

**Methods:**

A randomized controlled trial will be conducted. Patients to be included suffer from non-inflammatory degenerative joint disease of the hip, are aged between 18 and 80 and are admitted for primary cementless unilateral total hip arthroplasty. Patients in the metal-on-metal group will receive a cementless titanium alloy acetabular component with a cobalt-chromium liner and a cobalt-chromium femoral head varying from 38 to 60 mm. Patients in the metal-on-polyethylene group will receive a cementless titanium alloy acetabular component with a polyethylene liner and a 28 mm cobalt-chromium femoral head. We will assess acetabular bone mineral density by dual energy x-ray absorptiometry (DEXA), serum ion concentrations of cobalt, chromium and titanium, self reported functional status (Oxford hip score), physician reported functional status and range of motion (Harris hip score), number of dislocations and prosthetic survival. Measurements will take place preoperatively, perioperatively, and postoperatively (6 weeks, 1 year, 5 years and 10 years).

**Discussion:**

Superior results of large head metal-on-metal total hip arthroplasty over conventional hip arthroplasty have been put forward by experts, case series and the industry, but to our knowledge there is no randomized controlled evidence.

**Conclusion:**

This randomized controlled study has been designed to test whether large head metal-on-metal cementless total hip arthroplasty leads to less periprosthetic bone density loss and higher serum metal ion concentrations compared to 28 mm metal-on-polyethylene cementless total hip arthroplasty.

**Trial registration:**

Netherlands Trial Registry NTR1399

## Background

Painful osteoarthritis of the hip can be successfully treated by total hip arthroplasty (THA). Conventional total hip prostheses consist of a 28 mm metal head and a polyethylene cup. Polyethylene wear debris can however lead to osteolysis, bone loss, aseptic loosening and eventually failure of the implant, especially in high demand young patients [1]. Metal-on-metal (MM) total hip arthroplasty is an alternative to overcome polyethylene wear induced prosthetic failure. The MM wear rate is reported to be 20 to 100 times lower than conventional polyethylene wear rates, roughly 6 μm per year [2]. MM wear rate is also influenced by the size of the articulation and its clearance (i.e. the difference between the radius of the head and the shell): larger heads show lower wear rates provided they have a low clearance [3]. Another advantage of larger head sizes seems to be an increased range of motion and a reduced number of dislocations [4]. The main claim of metal-on-metal articulations is a reduction of wear and a subsequent lower incidence of periprosthetic osteolysis. Since osteolysis is implicated in the early phases of prosthetic loosening and failure, it is essential to accurately quantify periprosthetic osteolysis. Conventional radiology is not sensitive and accurate enough to detect small amounts of osteolysis, but dual energy x-ray absorptiometry (DEXA) is able to detect even small defects in the periprosthetic bone in the acetabulum [5]. In spite of the advantages of low wear and fewer dislocations, metal-on-metal hip prostheses increase systemic cobalt and chromium ion concentrations [6]. The long term effects of these ions are unknown, but concerns are hypersensitivity, mutagenicity and carcinogenicity [7].

The objective of this study is to conduct a randomized controlled trial to compare two cementless total hip arthroplasties: a conventional 28 mm metal-on-polyethylene articulation and a metal-on-metal large head articulation. We hypothesize that the large head metal-on-metal arthroplasties show less bone mineral density loss and higher serum metal ion concentrations (primary outcome parameters). We expect equal functional scores, greater range of motion, less dislocations, fewer periprosthetic radiolucencies and increased prosthetic survival with the MM articulation (secondary outcome parameters). The present paper reports on the design of the study.

## Methods

### Study design

A randomized controlled trial will be conducted and concealed allocation will be used to allocate patients to either metal-on-polyethylene or metal-on-metal cementless total hip arthroplasty. The randomization procedure is based on sequentially numbered opaque sealed envelopes, produced by an external institution not involved in the selection, clinical care and evaluation of the patients. The study design, procedures and informed consent are approved by the local Medical Ethical Committee (registration number 2005–42). The trial is registered in the Netherlands Trial Registry (NTR1399). Guidelines of the Consort Statement are followed [8].

### Study population

The study will be conducted at the Department of Orthopaedic Surgery of the Martini Hospital, which is a large teaching hospital in the city of Groningen, the Netherlands. Patients to be included suffer from non-inflammatory degenerative joint disease of the hip including osteoarthritis, avascular necrosis and traumatic arthritis, are aged between 18 and 80 and are admitted for primary cementless unilateral THA. Patients with active infection, revision arthroplasty, marked bone loss, and unwillingness or inability to follow instruction are excluded. Participation in the study is voluntary and informed consent is required. The inclusion period is planned from September 2006 to September 2009.

### Intervention

#### Metal-on-metal (MM)

Patients in the metal-on-metal group will receive a metal-on-metal articulation total hip arthroplasty, a cementless plasma sprayed porous coated titanium alloy acetabular component with a cobalt-chromium liner (M2a-Magnum™, Biomet) and a cobalt-chromium femoral head with a carbon concentration between 0.20% and 0.30%. The radial clearances of the articulations vary between 17.5 and 150 micrometers. The head sizes vary from 38 to 60 mm, depending on the shell sizes which range from 44 to 66 mm. The geometry of the patient determines the largest possible shell size and head size to be implanted.

#### Metal-on-polyethylene (MP)

Patients in the metal-on-polyethylene group will receive a metal-on-polyethylene total hip arthroplasty, a cementless plasma sprayed porous coated titanium alloy acetabular component (Mallory-Head^®^, Biomet) with a polyethylene liner (ArCom™, Biomet) and a 28 mm cobalt-chromium femoral head with a carbon concentration between 0.20% and 0.30%. In both the MM and MP groups the same cementless femoral component is used: a proximally plasma sprayed porous coated titanium alloy (Ti_6_Al_4_V) stem (Mallory-Head^®^, Biomet).

According to the surgeon's preference, a posterolateral or anterolateral surgical approach in lateral decubitus position is used. Antibiotic prophylaxis with a first-generation cephalosporin will be given preoperatively and during the first twenty-four hours intravenously. All patients will be treated postoperatively following a standardized protocol, in terms of analgesia and mobilization. As prophylaxis against thrombosis, oral anticoagulation by coumarin-derivate is given 6 weeks postoperatively.

### Measurements

In this study the following outcome parameters will be assessed: bone densitometry and serum metal ion concentration (primary outcome parameters), self reported functional status, physician reported functional status, range of motion, number of dislocations, radiographic evaluation and prosthetic survival (secondary outcome parameters). Measurements will take place preoperatively, perioperatively, and postoperatively (6 weeks, 1 year, 5 years and 10 years).

#### Bone densitometry

Bone mineral density (BMD) measurements will be performed using a dual energy x-ray absorptiometry (DEXA) scanner (Hologic Inc., Bedford, Mass., United States) in order to calculate bone density changes around the acetabular component. Four horizontal regions of interest (ROI) are defined, as suggested by Wilkinson [9]. In addition, an extra ROI is defined in the os ilium to serve as control. The manufacturer's metal removal software will be used. The contralateral normal hip will be scanned following a standard manufacturer's protocol to establish BMD in the femoral neck, trochanter, intertrochanteric, total hip and Ward's triangle sites.

#### Serum metal ion concentration

Serum ion concentrations for cobalt, chromium and titanium will be determined by venous blood sampling. Cobalt and titanium concentrations are analyzed by inductively coupled plasma mass spectrometry (ICP-MS; Agilent 7500 series, Agilent Technologies) and chromium is measured by grafite furnace atomic absorption spectrometry with Zeeman correction (GFAAS; Varian 220Z, Varian Inc.). The patients' sera may also be used to assess cytokine levels and effects of these ions on osteoblast cells.

#### Perioperative measurements

Surgical approach, surgical time and intra-operative blood loss are recorded. Perioperative complications will be registered, including hip dislocations.

#### Self-reported and physician reported functional status and range of motion

The validated Oxford self-rating questionnaire will be used to assess self reported functional status [10]. The validated Harris Hip Score is used to assess patient and physician reported functional status, as well as range of motion [11,12].

#### Radiographic evaluation

During every follow-up visit standard supine anteroposterior (AP) pelvic hip radiographs (with 115% magnification) will be taken. The AP radiographs at 6 weeks will serve as baseline, and will be compared to the X-rays 5 years and 10 years postoperatively. Radiographs are reviewed for presence of femoral radiolucent lines and scored according to the 7 zones described by Gruen et al. [13]. Peri-acetabular radiolucencies are assessed according to the three zones of De Lee and Charnley [14]. The scoring will be undertaken by an independent reviewer.

### Sample size

It is our hypothesis that large head metal-on-metal arthroplasties will show less bone mineral density loss and higher serum metal ion concentrations compared to the conventional 28 mm metal-on-polyethylene articulations. In order to detect a least clinical relevant difference in bone mineral density (BMD) of 0.25 g/cm^2 ^with a standard deviation of 0.4, 41 patients are needed in each group (alpha 0.05, power 0.80). Based on previous work with cemented THA we expect a drop-out rate of 10%, but we also expect conversion to cemented cups if adequate cementless fixation fails. We therefore aim to include 50 patients in each group. Comparable studies also used 50 patients in each group [15,16]. In order to detect a clinical difference of 2.5 μg/liter in serum metal ion concentration with a standard deviation of 1.8, 8 patients per group are needed (alpha 0.05, power 0.80). To compensate for patients withdrawn from the study, (the first) 15 patients will be included in each group. A comparable study used 10 patients in each group [17].

### Statistical analysis

The Statistical Package for the Social Sciences version 14.0 for Windows (SPSS Inc.) will be used. Group comparisons are based on intention-to-treat analysis. Non-parametric tests are used for comparisons of means within groups (Wilcoxon's Signed Ranks Test) and between groups (Mann-Whitney Test) if our expectation of a skewed distribution of Oxford and Harris Hip scores postoperatively is confirmed. Chi-square (Fisher's Exact) tests are employed for analyses of categorical variables. Cumulative implant survival is calculated by Kaplan-Meier time series (Mantel-Cox log rank test). A two-sided p-value of < 0.05 is assumed to be significant.

## Discussion

Superior results of large head metal-on-metal total hip arthroplasty over conventional hip arthroplasty have been put forward by experts, case series and the industry, but to our knowledge there is no randomized controlled evidence. This study will compare both arthroplasties. Periprosthetic bone density loss will be the main focus. In the short term we will be able to determine whether large head articulations increase clinical range of motion and reduce the number of dislocations. Furthermore, the trial will provide insight in short-term and long-term serum metal ion levels. A related research project will focus on the effects of metal ions on human osteoblast cells in vitro. This is important since the long-term risks of systemic metal ion exposure are unknown. Major pitfalls in orthopaedic surgery research have been the absence of a control group and the lack of randomization. This study overcomes both these drawbacks.

## Conclusion

This randomized controlled study has been designed to test whether large head metal-on-metal cementless total hip arthroplasty leads to less periprosthetic bone density loss and higher serum metal ion concentrations compared to 28 mm metal-on-polyethylene cementless total hip arthroplasty.

## Competing interests

Biomet Netherlands financed the bone densitometry measurements and the serum metal ion analysis. The authors did not receive any reimbursements, fees or salary for performing the study.

## Authors' contributions

WPZ designed the study and the data collection protocols, included and reviewed part of the patients, coordinated the trial, wrote the manuscript, and will analyze the data. NB included part of the patients and coordinated the trial. JJAMVR designed the study, included, operated and reviewed part of the patients. All authors read, edited and approved the final manuscript.

## Pre-publication history

The pre-publication history for this paper can be accessed here:


